# Perioperative Modulation of Left Ventricular Systolic Performance: A Retrospective Study on Ionized Calcium and Vitamin D in Cardiac Surgery Patients

**DOI:** 10.3390/jpm14080850

**Published:** 2024-08-10

**Authors:** Adrian Ștef, Constantin Bodolea, Ioana Corina Bocșan, Alexandru Achim, Nadina Tintiuc, Raluca Maria Pop, Aurelia Georgeta Solomonean, Alexandru Manea, Anca Dana Buzoianu

**Affiliations:** 1 Clinical Department of Anesthesia and Intensive Care, Heart Institute “Niculae Stancioiu”, University of Medicine and Pharmacy “Iuliu Hatieganu”, Motilor 19-21, 400001 Cluj-Napoca, Romania; 2 Department of Surgery, Discipline of Anesthesia and Intensive Care 2, University of Medicine and Pharmacy “Iuliu Hatieganu”, Victor Babes Nr. 8 Street, 400012 Cluj-Napoca, Romania; 3 Cardiology Department, Heart Institute “Niculae Stancioiu”, University of Medicine and Pharmacy “Iuliu Hatieganu”, Motilor 19-21, 400001 Cluj-Napoca, Romania; 4 Department of Pharmacology, Toxicology and Clinical Pharmacology, University of Medicine and Pharmacy “Iuliu Hatieganu”, Victor Babes Nr. 8 Street, 400012 Cluj-Napoca, Romaniaraluca_parlog@yahoo.com (R.M.P.);; 5 Cardiovascular Surgery Department, Heart Institute “Niculae Stancioiu”, University of Medicine and Pharmacy “Iuliu Hatieganu”, Motilor 19-21, 400001 Cluj-Napoca, Romania

**Keywords:** ionized calcium, vitamin D, ejection fraction, left ventricle systolic function, cardiac surgery, cardio-pulmonary bypass, open-heart surgery

## Abstract

**Background**: The perioperative impact of calcium and vitamin D on left ventricular (LV) performance during major cardiac surgery remains unexplored. We aimed to assess the relation of calcium and vitamin D measured at different time points with the LV ejection fraction (EF), and to investigate whether changes in EF correlate with postoperative outcomes. **Methods**: We enrolled 83 patients, in whom ionized calcium was measured before, during, and after surgery (until discharge), vitamin D preoperatively, and EF pre- and postoperatively at 24 h. The postoperative outcomes were cardiopulmonary bypass (CPB) time, aortic cross-clamp time, mechanical ventilation time, vasoactive inotropic score (VIS) (intraoperative, day 0, day 1), and ICU stay time. **Results**: The mean age was 64.9 ± 8.5 years, with 21 of the patients (25%) having an EF < 50%. The median change from preoperative to postoperative EF was −2.0 (−10.0–0.0) % (*p* < 0.001). At the baseline, the EF < 50% group had significantly lower preoperative vitamin D levels than the EF ≥ 50% group (*p* = 0.048). The calcium trend did not differ across the groups. Preoperative EF was significantly associated with CPB time (r = 0.22, *p* = 0.044) and aortic cross-clamp time (r = 0.24, *p* = 0.031). Postoperative EF was significantly and inversely associated with intraoperative VIS (r = −0.28, *p* = 0.009), VIS day 0 (r = −0.25, *p* = 0.020), VIS day 1 (r = −0.23, *p* = 0.036), and ICU length of stay (r = −0.22, *p* = 0.047). Finally, the change in ejection fraction was significantly and inversely associated with CPB time (r = −0.23, *p* = 0.037), aortic cross-clamp time (r = −0.22, *p* = 0.044), intraoperative VIS (r = −0.42, *p* < 0.001), VIS day 0 (r = −0.25, *p* = 0.024), mechanical ventilation time (r = −0.22, *p* = 0.047), and ICU length of stay (r = −0.23, *p* = 0.039). **Conclusions**: The fluctuations in perioperative ionized calcium levels were not associated with the evolution of LVEF, although preoperative vitamin D levels may affect those with low EF. Correspondingly, a reduced EF significantly impacted all the studied postoperative outcomes. Further investigation into biomarkers affecting cardiac inotropic function is warranted to better understand their significance.

## 1. Introduction

Low cardiac output syndrome, myocardial infarction, and in-hospital death are significant postoperative complications in cardiac surgery, impacting 8–12% of patients undergoing open-heart procedures [1]. It is crucial to identify the factors that can impact the aforementioned major cardiac and cardiovascular events (MACCEs). Biomarkers have become integral in the landscape of major cardiovascular surgery, offering insights into disease mechanisms, aiding in the diagnosis, and enhancing patient management and outcomes. The aim of biomarker identification in cardiovascular surgery is multifaceted. Primarily, it seeks to improve risk stratification, early detection of postoperative complications, and personalized treatment approaches. By understanding the biological underpinnings of cardiovascular diseases through biomarkers, clinicians can predict adverse events, monitor surgical success, and potentially discover new therapeutic targets [2].

Certain hormones and electrolytes must be in balance for healthy cardiac function [2]. The process of cardioplegia and cardiopulmonary bypass (CPB) leads to disruptions in electrolyte and hormone metabolism, and the transition from CPB to spontaneous heart function is purposely restricted to a short period of time [3]. To terminate CPB, specific cardiac agents, such as inotropes, are required to enhance cardiac function. The choice of inotropic and vasopressor medications depends on the patient’s cardiovascular condition and hemodynamic status [4]. Beta-adrenergic agonists like epinephrine and calcium are commonly used during weaning from CPB and restoration of the ejection fraction (EF) [5]. Calcium and vitamin D, crucial elements in maintaining and regulating normal heart function, play pivotal roles in cardiomyocyte contraction and expansion [5,6,7].

In the context of chronic heart failure, the exact role of calcium remains elusive. Wang et al. discovered a link between low serum calcium and left ventricular systolic dysfunction in patients with coronary artery disease (CAD) [8]. Other studies have suggested an independent association between high serum calcium levels and heart failure [9,10,11]. Within heart cells, structures such as sarcoplasmic reticulum Ca^2+^-ATPase (SERCA2a) and sarcolemmal Na+/Ca^2+^ exchanger (NCX1) play crucial roles in maintaining calcium homeostasis, but their functions and expressions in heart failure have yielded conflicting results [12].

Recent evidence suggests that both ionized calcium and vitamin D deficit are prevalent in cardiac surgical patients and are independently associated with the risk of MACCE [13,14,15,16]. Most of the existing data focused on vitamin D restoration, showing that perioperative vitamin D supplementation improves the postoperative outcomes after cardiac surgery [17,18,19]. On the other hand, no robust evidence exists regarding the safety and clinical efficacy of calcium salts in patients undergoing cardiac surgery [20]. Real-life data show that calcium salts are widely used in adult patients to support hemodynamics during CPB weaning. However, there is no widely accepted practice regarding the type of drug, optimal dose, and mode of administration, especially during bypass termination [21]. An exploration of these interconnected biomarkers within the clinical–pathophysiological context of cardiac performance, specifically left ventricular systolic function, would provide valuable insights into whether an imbalance in calcium or vitamin D levels might lead to adverse postoperative clinical outcomes.

The objective of this study was to investigate the potential impact of perioperative fluctuations in calcium or vitamin D levels on left ventricular EF, and to examine whether changes in EF correlate with adverse postoperative outcomes, including CPB time, aortic cross-clamp time, vasoactive inotropic score (VIS), and intensive care unit (ICU) length of stay.

## 2. Methods

This study, approved by the “Iuliu Hatieganu” University of Medicine Ethics Committee (approval number 259 on 28 September 2023), was conducted as a single-center, retrospective, single-arm, open-label, observational investigation. Considering the retrospective nature and minimal risk to participants, the Ethics Committee waived the need for individual consent. Two groups were stratified based on EF: EF < 50% and EF > 50%. EF measurements were consistently obtained before and 24 h after surgery by the attending cardiologist, utilizing the Simpson method during bedside transthoracic ultrasonography (TTE).

**Figure 1:**
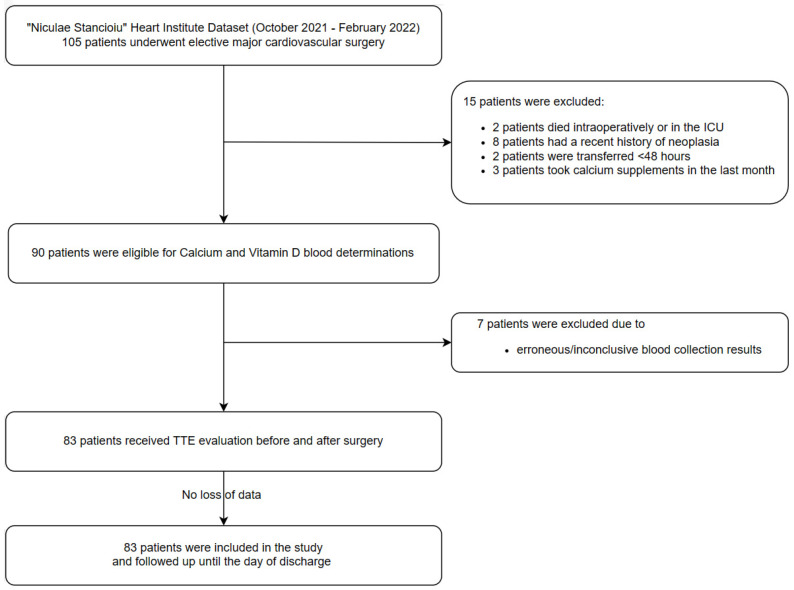
Study flow chart.

Inclusion criteria: all patients over 18 years old who underwent elective cardiovascular procedures requiring total anesthesia and cardiopulmonary bypass between 01 October 2021 and 28 February 2022 were enrolled. The study flow chart and design is presented in Figure 1. Twenty-two patients were excluded from this study. Exclusion criteria included urgent indications, unstable patients referred for major cardiovascular surgery, individuals with a history of hyperparathyroidism or active neoplasia, calcium or vitamin D supplements intake prior to hospitalization, inadequate blood collection protocols, inadequate TTE acquisitions, incomplete information for calculating the VIS score, and patients with a hospital length of stay less than 48 h (including those who expired or were transferred to other facilities). All the patients were followed up until the day of discharge.

Vitamin D levels were assessed 2 h prior to surgery. Ionized calcium levels were measured at predefined intervals: preoperatively, during CPB, immediately post-surgery (day 0), 24 h post-surgery (day 1), and upon discharge from the intensive care unit for patients with prolonged stays. All measurements were conducted from venous blood using the Biotek Microplate 50 TS washer (Agilent Technologies Inc., Santa Clara, CA, USA) and the 800 TS reader (Agilent Technologies Inc., Santa Clara, CA, USA). Vitamin D was determined using electrochemiluminescence, and ionized calcium was indirectly calculated from the value of serum calcium and total protein according to the following formula: Ca++ = (6 × Ca − PT/3):(PT + 6)^3^.

The clinical parameters were measured by two independent ICU physicians and were as follows: CPB time (minutes), aortic cross-clamp time (minutes), mechanical ventilation time (minutes), VIS intraoperative, day 0, and day 1, and ICU stay time (days). The VIS (a strong predictor of mortality and morbidity after cardiac surgery) was calculated with the following formula: VIS  =  dopamine dose [mcg/kg/min]  +  dobutamine [mcg/kg/min]  +  100 × epinephrine dose [mcg/kg/min]  +  10 × milrinone dose [mcg/kg/min]  +  10,000 × vasopressin [units/kg/min]  +  100 × norepinephrine dose [mcg/kg/min]), using the maximum dosing rates of vasoactive and inotropic medications (mcg/kg/min or IU/kg/min).

The primary outcome was a change in EF according to vitamin D and ionized calcium levels. The secondary outcome was a composite of postoperative clinical parameters: CPB time, aortic cross-clamp time, mechanical ventilation time, VIS (intraoperative, day 0, day 1), and ICU length of stay. For the patients included in the analysis, there was no loss of data.

### Statistical Analyses

The continuous variables underwent assessment for normality using the Shapiro–Wilk test and were presented as mean ± standard deviation or median (interquartile range) as deemed suitable. Categorical variables were expressed as frequencies and percentages. Groups were compared using the chi-squared test, Student’s *t*-test, Wilcoxon signed rank test, or Mann–Whitney U test, as appropriate. Spearman’s correlation coefficients with *p*-values were calculated for the associations of the ejection fraction with vitamin D, ionized calcium, and the outcomes. The Pearson correlation was used to calculate the linear relationship between calcium levels and LVEF before surgery and after surgery and the change in EF (delta EF). All analyses were completed using R Statistical Software (version 4.1.1, Foundation for Statistical Computing, Vienna, Austria).

## 3. Results

### 3.1. Study Population

The characteristics of the 83 included patients are summarized in Table 1.

**Figure 2:**
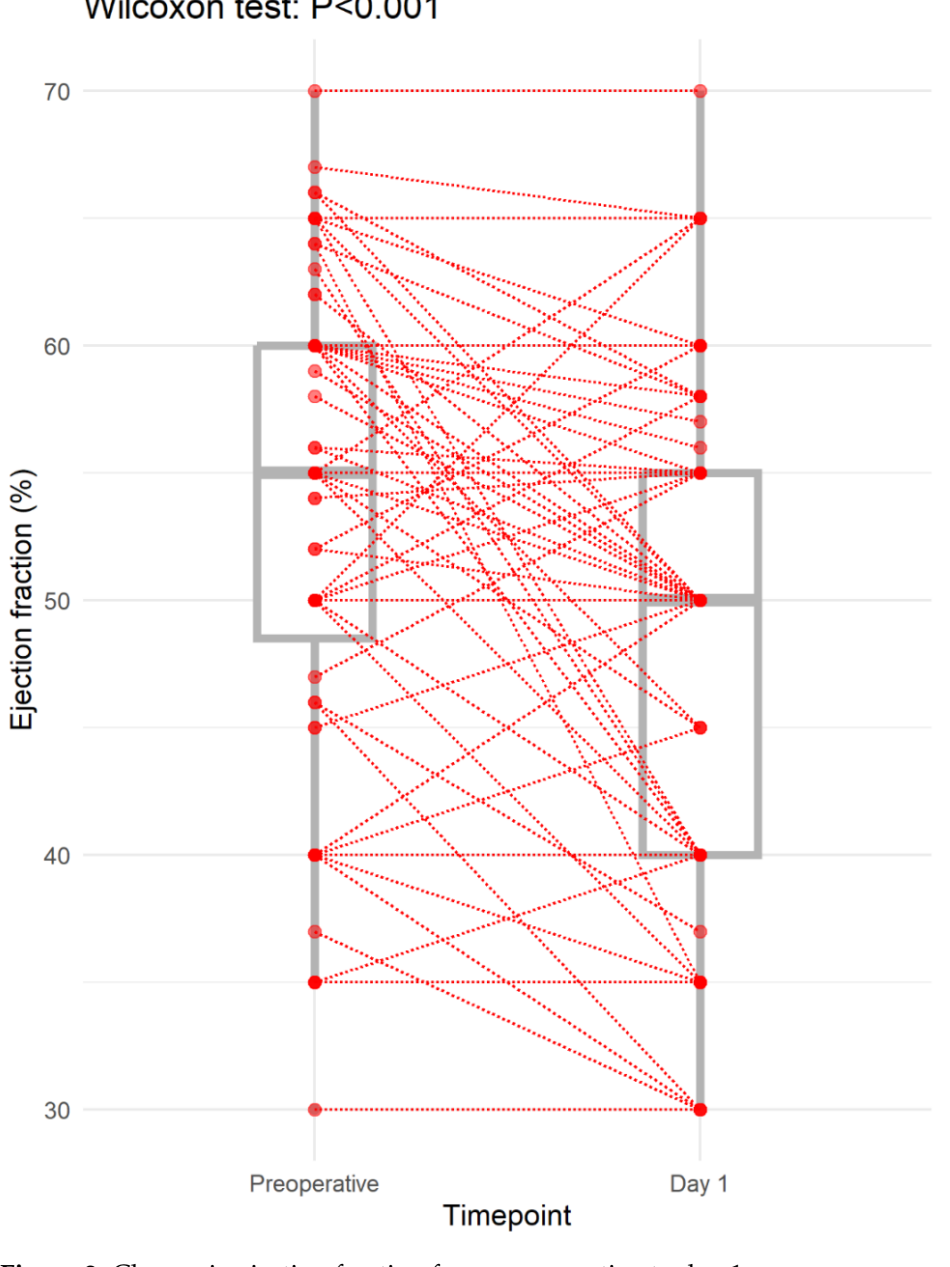
Change in ejection fraction from preoperative to day 1.

The mean age was 64.9 ± 8.5 years. The majority of procedures encompassed isolated CABG (N = 26, 31.3%), aortic valve procedures (N = 26, 31.3%), and mitral valve procedures (N = 12, 14.5%). The median preoperative vitamin D level was 15.8 (14.5–19.0) ng/mL. The levels of ionized calcium throughout the study period were 1.23 (1.20–1.25) mmol/L preoperatively, 1.14 (1.11–1.17) mmol/L during CPB, 1.20 (1.18–1.23) mmol/L at day 0, 1.21 (1.19–1.23) mmol/L at day 1, and 1.22 (1.20–1.25) mmol/L at discharge. There were no high ionized calcium alterations within the cohort, with a maximum value of 1.35 mmol/L found before surgery and with a significant drop-out during CPB, the lowest value being 1.01 mmol/L (*p* = 0.03), but with a similar behavior across the two groups (*p* = 0.4) (Figure 2).

The median CPB and aortic cross-clamp times were 100 min and 77 min, respectively. The median duration of mechanical ventilation was 3 h. Regarding hospitalization, the median lengths of stay in the ICU and hospital were 3 and 9 days, respectively. The median VIS showed a value of 4 intraoperatively, 3.5 on day 0, and 1.3 on day 1.

The median preoperative ejection fraction was 55.0 (48.5–60.0) %, with 21 (25.3%) of the patients having a perioperative ejection fraction of <50%. The median change from preoperative to postoperative ejection fraction was −2.0 (−10.0–0.0) % (Wilcoxon signed rank test: *p* < 0.001; Figure 2). There was no significant difference in the EF change across the two groups (*p* = 0.42).

**Figure 3:**
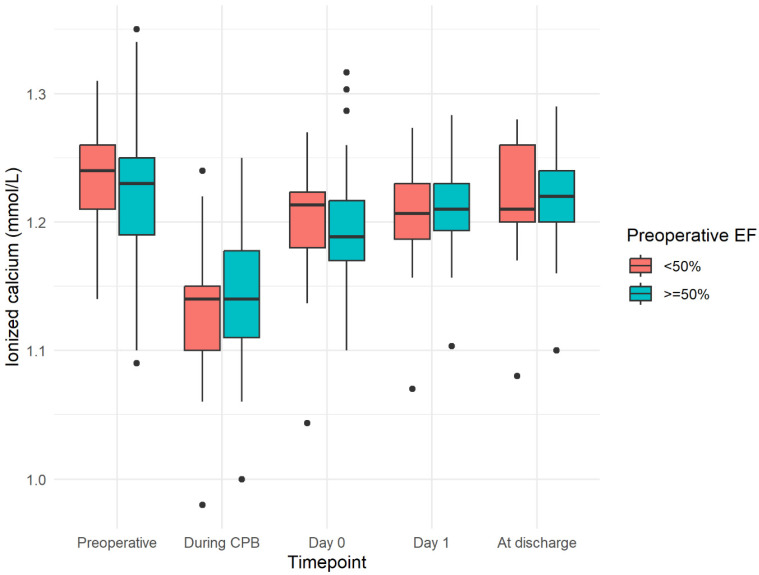
Trend in ionized calcium during the study period, according to preoperative ejection fraction (EF) group.

The subjects with preoperative ejection fraction <50% had significantly lower preoperative vitamin D levels than those with preoperative ejection fraction ≥50% (*p* = 0.048); at the same time, these individuals (the low EF patients) were correlated with a poorer clinical outcome, as demonstrated in the Spearman correlation found in the subsection below. Other characteristics did not differ significantly according to the preoperative ejection fraction group. Neither did the trend in calcium differ according to these groups (Figure 3).

### 3.2. Correlations of Ejection Fraction with Vitamin D, Ionized Calcium, and Outcomes

**Figure 4:**
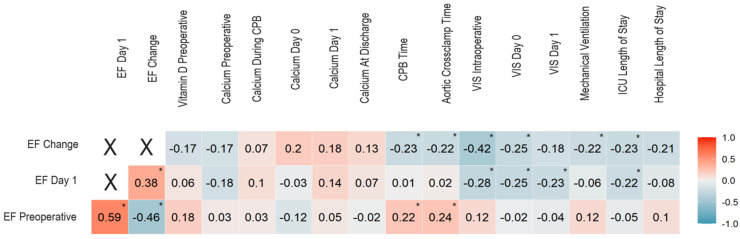
Spearman’s correlation coefficients for the associations of ejection fraction with vitamin D, ionized calcium, and outcomes. Correlation coefficients are given. Statistically significant correlation coefficients (*p* < 0.05) are indicated by *.

Figure 4 depicts the Spearman’s correlation coefficients for the associations of ejection fraction with vitamin D, ionized calcium, and outcomes. The preoperative ejection fraction was significantly associated with CPB time (r = 0.22, *p* = 0.044) and aortic cross-clamp time (r = 0.24, *p* = 0.031). The postoperative ejection fraction, i.e., at day 1, was significantly and inversely associated with intraoperative VIS score (r = −0.28, *p* = 0.009), VIS score at day 0 (r = −0.25, *p* = 0.020), VIS score at day 1 (r = −0.23, *p* = 0.036), and ICU length of stay (r = −0.22, *p* = 0.047).

**Figure 5:**
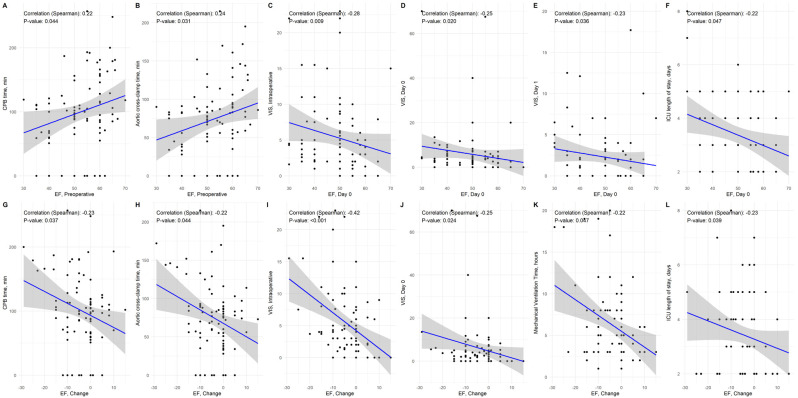
Significant correlations between EF and outcomes (preoperative EF sub-figures (**A**,**B**), postoperative EF subfigures (**C**–**F**), and change in EF subfigures (**G**–**L**)). EF, ejection fraction; CPB, cardiopulmonary bypass; VIS, vasoinotropic score.

Finally, the change in ejection fraction (with a negative value corresponding to a decrease from preoperative to postoperative values) was significantly and inversely associated with CPB time (r = −0.23, *p* = 0.037), aortic cross-clamp time (r = −0.22, *p* = 0.044), intraoperative VIS score (r = −0.42, *p* < 0.001), VIS score at day 0 (r = −0.25, *p* = 0.024), mechanical ventilation time (r = −0.22, *p* = 0.047), and ICU length of stay (r = −0.23, *p* = 0.039). The significant correlations are summarized in Figure 5.

**Figure 6:**
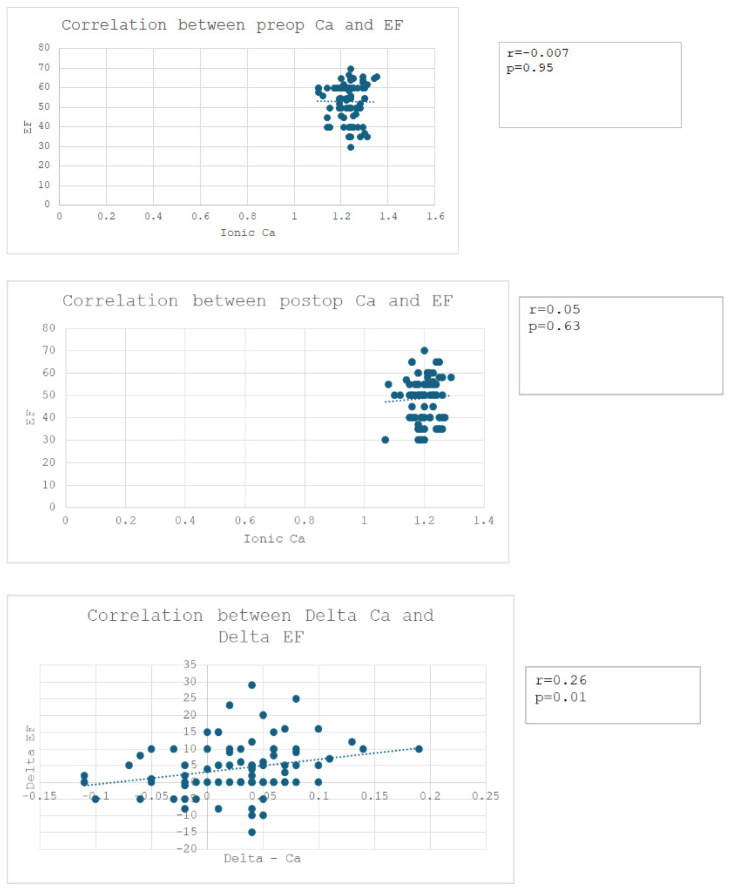
Correlations between preoperative (first panel), postoperative (middle panel), and differences in ejection fraction and calcium levels (last panel). There is no linear relationship between the calcium levels and LVEF before and after surgery, but the decrease in calcium values is weakly to moderately directly proportional to the decrease in LVEF values (r = 0.26, *p* = 0.01).

Regarding the Pearson correlation, there were no statistically significant correlations observed between the preoperative calcium levels and preoperative EF, nor between the postoperative calcium levels and postoperative EF (Figure 6). However, a weak-to-moderate statistically significant linear correlation (r = 0.26, *p* = 0.01) was demonstrated between the differences in the preoperative and postoperative values for these factors (Figure 6).

## 4. Discussion

The main findings of our study indicate that (1) fluctuations in perioperative ionized calcium levels did not affect left ventricular systolic function before and after major cardiovascular surgery requiring CPB. However, (2) there was a significant decrease in preoperative vitamin D levels among individuals with an EF of less than 50% (correlating further with a worse clinical progression). In terms of clinical significance, (3) a reduced EF (including preoperative, postoperative, and the change in EF) was correlated with all the studied clinical outcomes, including a longer CPB and aortic clamp time, a longer duration of mechanical ventilation, higher VIS, and a longer stay in the ICU. The change in EF was the only parameter which correlated with all the outcomes. It is necessary to acknowledge that the EF change was similar across the two groups; therefore, the outcomes were not driven by a potential lower change within the low EF group. Despite calcium’s traditional role as an inotropic agent, our findings suggest that its significance may not be as pronounced in cases where calcium levels remain within normal ranges (our cohort did not contain severe hypocalcemia cases <1.00 mmol/L). Furthermore, our study highlights the potential impact of vitamin D on post-surgical outcomes, drawing parallels with its role in chronic heart failure.

Our findings align with previously established data indicating that open-heart surgery triggers acute stress, which has been shown to negatively impact circulating concentrations of 25-hydroxyvitamin D (25(OH)D) [14,18,19,22]. The global prevalence of low vitamin D levels constitutes a significant health concern, with associations noted between low vitamin D and increased rates of all-cause and cardiovascular mortality. Notably, low levels of vitamin D are frequently observed preoperatively in patients undergoing cardiac surgery and are independently linked to the occurrence of major adverse cardiac and cerebrovascular events during hospitalization, as well as mortality in the months following cardiac surgery [23]. Further data showed that perioperative vitamin D supplementation protects against the immediate decrease in plasma 25(OH)D induced by open-heart surgery [17,18,19]. In fact, a recent systematic review comprising eight randomized controlled trials could show that six (75%) studies were found to be in favor of improvement in postoperative VIS, ICU stay, postoperative atrial fibrillation, IL-10 levels, or fewer cardiac adverse events [24]. Braun et al. found that 67.5% of patients had serum vitamin D levels below 60 nmol/L prior to cardiothoracic surgery [23]. The contractile properties of cardiac cells are primarily governed by the direct interaction between calcium and the contractile proteins, actin and myosin, as well as the intracellular handling of calcium. Additionally, extracellular calcium homeostasis, influenced by vitamin D levels, affects intracellular calcium and can indirectly impact cardiac cell contractility. Our study design was created from this concept, although to date only experimental data have shown that mice blocked for the vitamin D receptors developed myocardial hypertrophy and dysfunction, and there is still a substantial discrepancy between the outcome of experimental studies and clinical intervention trials [25]. There are a number of factors that influence/modulate circulating levels of vitamin D, including different geographic latitudes, skin pigmentation, availability of vitamin D food sources, age, sex, cultural habits, and lifestyle [26]. Furthermore, hypoparathyroidism, severe kidney disease, and liver insufficiency can affect serum 25-OH vitamin D levels [27]. Excessive intake of vitamin D has been shown to cause vitamin D toxicity, which can lead to anorexia, weight loss, polyuria, and heartbeat irregularities [28].

The effects of calcium on left ventricular function early after cardiopulmonary bypass come from old studies which measured the cardiac index invasively and acutely (immediately after surgery) [29,30]. To our knowledge, studies investigating calcium values measured at different time points which could correlate with the evolution of the EF at least 24 h after surgery do not exist. The reasons for our differing results could be either the characteristics of our studied cohort (stable, elective patients, disease severity, small sample size, small number of patients with EF below 25%, etc.), the relatively stable ionized calcium values (with no major fluctuations), the time point of sample collection, or the calcium supplementation intervention. It should also be noted that there was no consensus on the management of ionized calcium disorder in ICU. The authors believe future research should focus on postoperative cardiac performance biomarkers (negative or positive), with emphasis on severe hypocalcemia or vitamin D deficit, following clinical outcomes in patients who received correction. Our results underline the significance of left ventricular function, a physiological condition that should not be taken “as is”, but rather as a target for improvement in the initial days following cardiopulmonary resuscitation. Notably, the crucial role of mechanical support devices in bridging therapy was not addressed, but it is of equal importance [31]. Nevertheless, calcium and vitamin D remain two important drivers for cardiovascular performance and a more careful humoral screening could lead to a faster recovery of the cardiac patient. The application of these biomarkers extends beyond risk assessment to personalized medicine. By integrating biomarker data with clinical parameters, tailored perioperative care plans can be developed, optimizing surgical timing, anesthesia management, and postoperative care. This approach not only enhances patient safety and outcomes but also contributes to the efficient allocation of healthcare resources.

The authors also believe that the association of a lower EF with all perioperative clinical parameters should be interpreted with caution, as these correlations may have associative relationships rather than causative ones—i.e., a more morbid population (with a lower EF) could have a more unfavorable evolution after surgery. It is well established that patients with low LVEF face elevated risks of postoperative complications and mortality following cardiac procedures, with low EF serving as a key predictor of unfavorable outcomes, incorporated into all existing scoring systems [32,33]. Indeed, low preoperative LVEF is linked to a range of postoperative complications, including, but not limited to, low cardiac output syndrome, requirement for inotropic support, acute renal failure, lung congestion, pneumonia, atrial fibrillation, stroke, sepsis or endocarditis, and deep sternal wound infection, as well as bleeding necessitating reoperation and gastrointestinal bleeding [34,35,36]. In addition to these findings, our study introduces the concept of EF change, or “EF behavior”, which exhibited the most robust correlations with the outcomes, comprising two-thirds of all significant correlations, surpassing the predictive power of preoperative EF alone.

The variance in CPB time likely stems from an extended reperfusion phase following the release of the aortic cross-clamp, which is particularly evident in patients exhibiting severely impaired left ventricular function [37]. One study showed that, to minimize the occurrence of unfavorable adverse outcomes, it is recommended that the CPB/graft time and cumulative CPB time be kept below 56 min and 180 min, respectively [38]. Nonetheless, numerous surgeons opt to employ CPB due to the frequent occurrence of hemodynamic instability, ventricular arrhythmia-induced hypotension, or cardiac arrests, especially within CABG procedures. A speculation exists regarding the potential exacerbation of myocardial damage in patients with compromised left ventricles as a result of extracorporeal circulation. In our analysis, the CPB and aortic cross-clamp time were correlated mainly with the preoperative EF and the change in EF. On the other hand, the postoperative EF showed a significant correlation primarily with the VIS scores and the length of stay in the ICU. These factors forecasted the requirement for increased inotropic support among this cohort, linked with a prolonged duration of ICU stay.

This study has a few limitations. The single-center nature and small sample size (including the small number of patients in the low EF group) precluded definite conclusions from being drawn and meant that our findings should be seen as hypothesis-generating. The observational nature was also predisposed to residual confounding despite having accounted for a large number of confounders by means of multivariable adjustments. These may include other factors that may influence a lower EF after cardioplegia and CPB, such as myocardial stunning and reperfusion injury, especially since the control EF was measured 1–2 days after, and not later, or factors that may have influenced vitamin D or calcium levels, such as the chronic medication (with an impact on blood electrolytes), which was not taken into account. The prevalence of chronic kidney disease (comorbidity with a heavy influence on both vitamin D and ionized calcium levels) was similar across the two groups. The significant difference in CABG, with more procedures in the low EF group, could have influenced the evolution and recovery of EF in these patients, implicitly affecting the outcomes. Moreover, mitral insufficiency was not quantified—an important factor that could have affected LV systolic function, although the EF < 50% and >50% groups had similar clinical characteristics. Another significant limitation is that the postoperative levels of vitamin D were not assessed; a correlation with the preoperative values would have brought further insights into vitamin D behavior in patients with different EFs. Lastly, the strong correlations of low EF with clinical outcomes could also be caused by the worse evolution of these patients due to an intraoperative myocardial injury; thus, a longer hospitalization period, etc., would be expected, although our study showed that the trend is maintained with regard to both the preoperative EF and the change in EF.

In conclusion, the identification and application of biomarkers in major cardiovascular surgery are pivotal in advancing patient care. While traditional biomarkers, such as inflammatory markers and cardiac troponins, have been well documented in predicting surgical risk and guiding clinical decision making, the roles of calcium and vitamin D are also gaining recognition. Dysregulation of calcium levels can lead to significant perioperative complications, including arrhythmias and myocardial ischemia. Similarly, vitamin D, which is essential for calcium homeostasis, has been associated with cardiovascular health. Deficiencies in vitamin D have been linked to increased risk of cardiovascular diseases and adverse surgical outcomes. Current data indicate that the monitoring and correcting of calcium and vitamin D levels perioperatively can improve patient outcomes by reducing complications and enhancing recovery [39,40,41]. Integrating the management of calcium and vitamin D with other biomarker strategies enhances the precision and efficacy of cardiovascular surgical interventions, fostering a more holistic approach to patient care. As research continues to evolve, a comprehensive biomarker panel that includes both traditional biomarkers and calcium and vitamin D will be crucial in advancing the field of cardiovascular surgery.

## 5. Conclusions

The evolution of left ventricular systolic function before and after major cardiovascular surgery requiring CPB was not influenced by the fluctuations of perioperative ionized calcium values, while vitamin D may play a role in those with low EF. On the other hand, a lower EF was associated with all the studied clinical outcomes (CPB time, aortic clamp time, mechanical ventilation time, VIS, and ICU length of stay). The importance of biomarkers with an effect on cardiac inotropic function deserves further research.

## Figures and Tables

**Table 1:** Population and surgical procedure characteristics as divided per preoperative low EF group (cut-off < 50%) and normal EF (cut-off ≥ 50%) group. BMI, body mass index; COPD, chronic obstructive pulmonary disease; CABG, coronary artery bypass grafting; ASD, atrial septal defect; CPB, cardiopulmonary bypass; ICU, intensive care unit; VIS, vasoinotropic score.

Variable	All (N = 83)	EF < 50% (N = 21)	EF ≥ 50% (N = 62)	*p*-Value
Age	64.9 ± 8.5	64.0 ± 11.1	65.2 ± 7.5	0.633
BMI	28.4 (25.2–31.6)	27.0 (23.7–30.4)	29.1 (25.4–31.8)	0.150
Hypertension	47 (56.6%)	12 (57.1%)	35 (56.4%)	0.632
Diabetes Mellitus	27 (32.5%)	8 (38%)	19 (30.6%)	0.110
Chronic Anemia	16 (19.3%)	4 (19%)	12 (19.3%)	0.238
COPD	14 (16.9%)	4 (19%)	10 (16.1%)	0.192
Chronic Kidney Disease	23 (27.7%)	6 (28.6%)	17 (27.4%)	0.654
Procedure				0.454
Isolated CABG	26 (31.3%)	11 (52.4%)	15 (24.2%)	0.016
Aortic valve procedure	26 (31.3%)	6 (28.6%)	20 (32.3%)	0.753
Mitral valve procedure	12 (14.5%)	1 (4.76%)	11 (17.7%)	0.144
Complex valve procedure	4 (4.82%)	1 (4.76%)	3 (4.84%)	0.989
CABG + valve procedure	5 (6.02%)	1 (4.76%)	4 (6.45%)	0.779
Bentall procedure	3 (3.61%)	1 (4.76%)	2 (3.23%)	0.744
Aortic valve and ascending aorta repair	3 (3.61%)	0 (0.00%)	3 (4.84%)	1.000
Aortic aneurysm repair	2 (2.41%)	0 (0.00%)	2 (3.23%)	1.000
ASD correction	2 (2.41%)	0 (0.00%)	2 (3.23%)	1.000
Atrial fibrillation	13 (15.7%)	1 (4.76%)	12 (19.4%)	0.168
Pacemaker	2 (2.41%)	1 (4.76%)	1 (1.61%)	0.444
Vitamin D Preoperative, ng/mL	15.8 (14.5–19.0)	15.0 (14.4–16.3)	16.1 (14.6–20.1)	0.048
Calcium Preoperative, mmol/L	1.23 (1.20–1.25)	1.24 (1.21–1.26)	1.23 (1.19–1.25)	0.386
Calcium During CPB, mmol/L	1.14 ± 0.05	1.13 ± 0.06	1.14 ± 0.05	0.403
Calcium Day 0, mmol/L	1.19 ± 0.05	1.20 ± 0.05	1.19 ± 0.05	0.518
Calcium Day 1, mmol/L	1.21 (1.19–1.23)	1.21 (1.19–1.23)	1.21 (1.19–1.23)	0.765
Calcium At Discharge, mmol/L	1.22 (1.20–1.25)	1.21 (1.20–1.26)	1.22 (1.20–1.24)	0.804
CPB time, min	100 (67.5–128)	100 (59.0–113)	101 (77.2–154)	0.154
Aortic cross-clamp time, min	77.0 (47.0–92.0)	75.0 (34.0–84.0)	77.0 (55.0–104)	0.178
Mechanical ventilation, hours	5.00 (3.00–8.00)	4.00 (3.00–8.00)	5.00 (3.00–8.00)	0.630
ICU length of stay, days	3.00 (2.00–4.00)	3.00 (2.00–4.00)	3.00 (2.00–4.00)	0.652
Hospital length of stay, days	9.00 (8.00–12.0)	8.00 (8.00–12.0)	9.00 (8.00–11.8)	0.526
VIS, intraoperative	4.00 (2.00–7.55)	3.00 (2.00–6.25)	4.00 (2.00–7.80)	0.821
VIS, day 0	3.50 (0.00–6.05)	3.90 (2.00–7.00)	3.25 (0.00–5.88)	0.536
VIS, day 1	1.30 (0.00–3.38)	2.20 (0.00–4.00)	1.23 (0.00–3.00)	0.205

## Data Availability

The raw data supporting the conclusions of this article will be made available by the authors, without undue reservation.

## Abbreviations

Major adverse cardiac and cerebrovascular events, MACCE; cardiopulmonary bypass, CPB; ejection fraction; EF; left ventricular, LV; vasoactive inotropic score, VIS; intensive care unit, ICU; transthoracic echocardiography, TTE.
